# Healthcare Resource Utilization, Treatment Costs, and Mortality in Patients with Malignancies or Transplantation Who Develop Invasive Aspergillosis

**DOI:** 10.3390/jof11090657

**Published:** 2025-09-06

**Authors:** Thomas J. Walsh, Craig I. Coleman, Melissa Johnson, Belinda Lovelace, Barbara D. Alexander

**Affiliations:** 1 Departments of Medicine and Microbiology & Immunology, University of Maryland School of Medicine, Baltimore, MD 21201, USA; 2 Center for Innovative Therapeutics and Diagnostics, Richmond, VA 23220, USA; 3 Department of Pharmacy Practice, School of Pharmacy, University of Connecticut, Storrs, CT 06269, USA; 4 Division of Infectious Diseases & International Health, Duke University, Durham, NC 27710, USA; 5 Health Economics and Outcomes Research, F2G, Inc., Princeton, NJ 08540, USA; belinda.lovelace@f2g.com

**Keywords:** invasive aspergillosis, malignancy, transplantation, healthcare utilization, mortality

## Abstract

**Objectives**: Invasive aspergillosis (IA) poses significant risks to patients with malignancies or transplantation; however, estimates of burden-of-illness in patients with IA are sparse. We sought to assess in-hospital and outpatient healthcare resource utilization, all-cause treatment costs, and mortality in patients admitted with IA with hematologic or non-hematologic malignancies, bone marrow transplant/hematopoietic cell transplantation (BMT/HCT), or solid organ transplantation (SOT). **Methods**: This claims study utilized United States IQVIA data. Adults admitted for IA were identified by diagnosis codes during the patient selection period (October 2015–November 2022). IA patients were stratified into cohorts including recent hematologic or non-hematologic malignancies, or a history of BMT/HCT or SOT. We assessed hospital and intensive care unit (ICU) length-of-stay (LOS), all-cause index hospital treatment costs, and inpatient mortality or need for hospice in each cohort, as well as the need for re-admission and total treatment costs for up to six-months after admission, and all-cause mortality at end of study follow-up. **Results**: Among 1190 patients admitted for IA, 317 had hematologic malignancies, 155 non-hematologic malignancies, 133 BMT/HCT and 173 SOT. Across these cohorts, IA was associated with protracted (median LOS = 12–18 days; ICU LOS = 10–13 days) and costly (median = USD 79,058–USD 172,342) index hospitalizations ending in death or hospice in 28.1% (89/317) to 36.1% (48/133) of patients. Among those surviving to discharge, between 53.1% (34/64) and 63.4% (97/153) were re-admitted within six months. Total median treatment costs at six months ranged from USD 213,378 to USD 397,857. All-cause mortality was 33.6% (52/155) to 40.6% (54/133) at end of study follow-up. **Conclusions**: Hospitalizations for IA in patients with malignancies or transplantation are long, costly, and end with readmission, hospice, or death in more than one-third of patients.

## 1. Introduction

Invasive aspergillosis (IA) is a serious mold disease caused by *Aspergillus* spp. [1] that poses significant risks to patients with malignancies or transplantation. *Aspergillus* spp. has been designated by the World Health Organization (WHO) as one of four critical priority fungal pathogens posing the greatest public health threat [2]. The annual global incidence of acute and chronic forms of invasive aspergillosis is estimated to exceed 2.1 million people [3]. Within the United States (US), IA accounts for more than 8000 hospitalizations per year [4].

Healthcare resource utilization (HCRU) and costs associated with treating IA can be substantial and result from intensive diagnostic testing, specialized care, supportive treatments for managing its complications, as well as underlying comorbidities, and costly antifungal therapeutics [4,5,6,7]. However, estimates of the burden-of-illness across the spectrum of care of managing “at-risk” IA patients with malignancies or transplantation are sparse.

We therefore sought to assess all-cause HCRU, total treatment costs, readmissions, and all-cause mortality or need for hospice for up to six-months after hospitalization for IA among patients with hematologic or non-hematologic malignancies, or a history of bone marrow transplant/hematopoietic cell (BMT/HCT) or solid organ transplantation (SOT).

## 2. Methods

This was a retrospective, observational cohort study that utilized nationwide US claims data from IQVIA’s New Data Warehouse [8]. This data set includes deterministically linked data from professional fee and prescription claims data and IQVIA’s hospital charge data master (CDM) database for facilities across the US. These data contain approximately one billion professional fee claims/year (representing >870,000 practitioners/month) with diagnoses, procedures, and office-administered medications; ~1.6 billion retail or mail-order prescription claims representing dispensed prescriptions for approximately 85% of all pharmacies; and records from >450 hospitals, including inpatient and outpatient encounters within a facility with medication, procedure, diagnosis, and charge data for the entire stay.

Adults admitted for IA (index date) were identified based on International Classification of Diseases-10th-Revision (ICD-10) codes (B44.0, B44.1, B44.7) in any position during the patient selection period of October 2015–November 2022. The first hospitalization for IA during the patient selection period was utilized, and the admission date for this IA hospitalization was defined as the index date (time 0). Data within the database from January 2010 to the index date minus one day for each patient were used to assess patient baseline comorbidities (details regarding the billing codes used are available from the corresponding author upon request).

In order to be included within the study, patient data needed to link to the professional fees and pharmacy claims data during the study period, have evidence of any systemic antifungal therapy (any combinations of triazoles, polyenes, or echinocandins) for ≥3 days during the hospitalization, have activity denoted as ≥1 medical claim in the professional fee data set and ≥1 pharmacy claim in the pharmacy claim data set during the six-month baseline period, and exhibit pharmacy stability (consistent reporting of data from the pharmacy most frequently visited by the patient during each month of the baseline period). The only exclusion criterion applied was evidence of non-IA invasive fungal infection during the index hospitalization.

Patients with IA were stratified into ICD-10 defined cohorts based on presence of hematologic (C81–C96) or non-hematologic malignancies (C00–C80) during a baseline period of six-months prior to the index date. Patients were stratified into ICD-10 defined cohorts of BMT/HCT (Z94.81, Z94.84, T86.0x, T86.5) or SOT (Z94.0–Z94.7, Z94.82, Z94.83, Z94.89, T86.1x–T86.9x) based upon a prior history of, or complication due to, BMT/HCT or SOT identified during a baseline period spanning January 2010 to the index date. Patients were allowed to fall into more than one cohort, if applicable, and counted in both (not mutually exclusive). Other comorbidities were identified using corresponding billing codes (available from the corresponding author upon request).

All results were determined separately for each of the four at-risk cohorts of interest. Median and interquartile range (IQR) all-cause total HCRU (total hospital and intensive care unit (ICU) length-of-stay (LOS)) and all-cause total costs in 2023 USD during index hospitalization were assessed. All-cause re-admission at one- and six-month post-discharge, all-cause cumulative total hospitalization and outpatient costs (office visits, emergency department visits, pharmacy, laboratory, pathology, and radiology), up to six months after the index date, and all-cause mortality through end of study follow-up also were assessed. To be included in the post-index IA admission analyses, patients were required to have at least the duration of follow-up of that outcome, i.e., only patients with at least six months of post-index admission follow-up data were included in the estimation of six months re-admission and cost analyses. As only charges were available in the data set, a national cost-to-charge ratio was applied to charges to convert them into costs. All analyses were performed in SAS version 9.4 (SAS Institute Inc., Cary, NC, USA).

The data were accessed in compliance with the Health Insurance Portability and Accountability Act (HIPAA). Institutional review board approval was not required for this retrospective analysis of deidentified secondary data. The data utilized are available only by license through IQVIA.

## 3. Results

### 3.1. Overall Population

**Figure 1:**
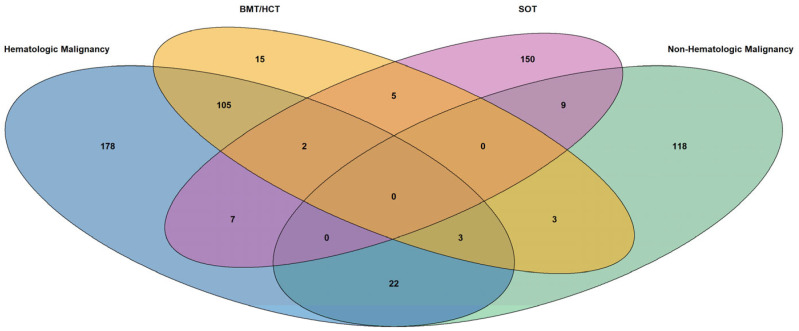
Venn diagram illustrating overlap between study cohorts. BMT/HCT = bone marrow transplant/hematopoietic cell transplant; SOT = solid organ transplant. The blue oval represents counts of patients with hematologic malignancy, the orange oval represents counts of patients with bone marrow or hemopoietic cell transplantation, the purple oval represents counts of patients with solid organ transplantation, and the green oval represents counts of patients with non-hematologic malignancy. Overlapping patient populations between cohorts are shaded grey.

**Figure 2:**
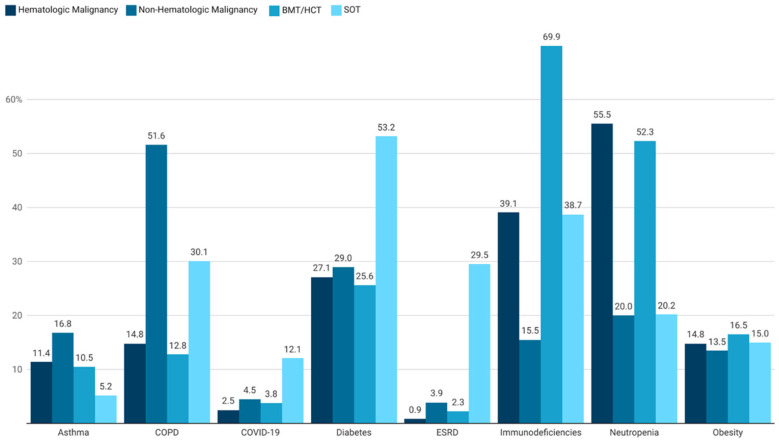
Baseline comorbidities of patients with malignancies or transplantation that developed invasive aspergillosis *^,†^. BMT = bone marrow transplant; HCT = hematopoietic cell transplant; COPD = chronic obstructive pulmonary disease; COVID-19 = coronavirus-19; ESRD = end-stage renal disease; SOT = solid organ transplant. * The hematologic malignancy, non-hematologic malignancy, BMT/HCT, and SOT cohorts are not mutually exclusive. ^†^ Incudes comorbidities in which at least one cohort had a >10% incidence. Figure created with Datawrapper (Datawrapper GmbH, Berlin, Germany; https://www.datawrapper.de/, accessed on 5 September 2025).

We identified 1190 patients in total with an IA admission, with 317 having hematologic malignancy, 155 having non-hematologic malignancy, 133 having BMT/HCT and 173 having SOT (Table 1, Figure 1). In each of these cohorts, males and those of older age (45 years or greater) predominated (Table 2). Patient admissions from the West and Southern regions of the US were more common. Medicare and commercial insurers were most prevalent across all cohorts. Comorbidities of these patient cohorts are depicted in Figure 2. Pre-exposure triazole use was common in all cohorts in the six months prior to index IA admission [55.8% (177/317) of patients with hematological malignancies, 30.3% (47/155) of those with non-hematologic malignancies, 71.4% (95/133 with BMT/HCT and 43.4% (75/173) with SOT]. Inpatient mortality exceeded 25% for all four cohorts, with all-cause mortality over the study period of more than 33%, and readmission at 1 month ranging from 13.6% to 26.1%. Six-month readmission rates were more than 50% for all cohorts.

### 3.2. Hematologic Malignancies

**Figure 3:**
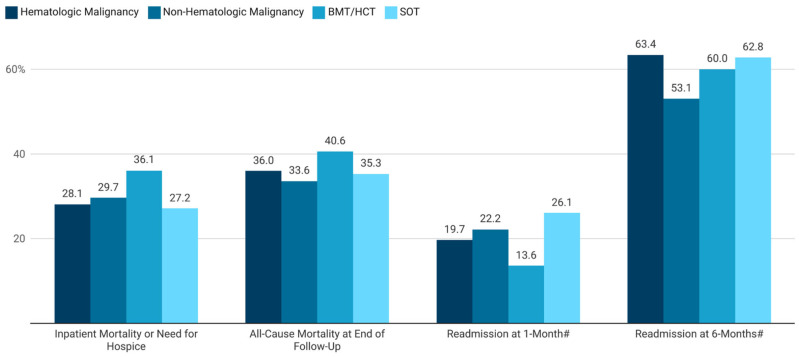
Inpatient mortality or need for hospice, all-cause mortality at end of follow-up, and readmission at 1- and 6-months post-hospitalization ^†^ in patients with history of malignancies or transplantation that developed invasive aspergillosis *. * The hematologic malignancy, non-hematologic malignancy, BMT/HCT, and SOT cohorts are not mutually exclusive. ^†^ Among patients with at least that duration of follow-up. # Among patients with at least that duration of follow-up. Figure created with Datawrapper (Datawrapper GmbH, Berlin, Germany; https://www.datawrapper.de/, accessed on 5 September 2025).

Among the 317 patients with hematologic malignancies, 82% (260/317) had leukemia. Common baseline comorbidities found in this cohort included neutropenia (55.5%), immunodeficiencies (39.1%), and diabetes mellitus (27.1%). These patients were found to have protracted total and ICU median LOSs (18 and 12 days, respectively), and costly hospitalizations (USD 151,489) that were associated with an inpatient mortality or need for hospice in 28.1% (89/317) of patients (Figure 3, Table 3). Nearly 20% (51/259) had an all-cause 30-day re-admission rate and 63.4% (97/153) were re-admitted by six months. By the end of follow-up, all-cause mortality was 36.0% (114/317) with a median time to death of 41 days.

### 3.3. Non-Hematologic Malignancies

Patients with non-hematologic malignancies who were admitted for IA (N = 155) most frequently had cancers of the lungs, trachea, or bronchi (54.2%, 84/155), followed by breast, urinary tract, and colorectal neoplastic disease (prevalence all ≥11.0%). Common baseline comorbidities in this cohort included chronic obstructive pulmonary disease (COPD) (51.6%), diabetes mellitus (29.0%), and neutropenia (20.0%). Median total and ICU LOSs were 12 and 10 days, respectively, and total index hospital costs were USD 79,058. One- and six-month re-admissions occurred in 22.2% (24/198) and 53.1% (34/64), respectively. One-third of this cohort (52/155) had died by the end of follow-up with a median time to death of 20 days.

### 3.4. BMT/HCT Recipients

BMT/HCT was observed in 133 of the IA-admitted patients. Immunodeficiencies and neutropenia were present in 52.3% and 69.9% of patients in this cohort. The median total and ICU LOSs for BMT/HCT recipients were 18 and 12 days, respectively, while 36.1% either died during hospitalization or were transferred to hospice. In those surviving to discharge, 13.6% (15/110) and 60.0% (36/60) were readmitted for any cause at one and six months, with total six-month healthcare costs of USD 389,296. By the end of follow-up, 40.6% of BMT/HCT recipients died with a median time to death of 51 days.

### 3.5. SOT Recipients

Within the cohort of all patients admitted for IA, 173 were SOT recipients. Lung and kidney were the most common transplanted organs. Diabetes mellitus (53.2%), immunodeficiencies (38.7%), COPD (30.1%), and neutropenia (20.2%) were the most frequent comorbidities. The median total and ICU LOSs for SOT recipients were 15 and 13 days, respectively, with 27.2% either dying during index admission or being transferred to hospice. In those surviving to discharge, 26.1% (36/138) and 62.8% (59/94), respectively, were re-admitted for any cause at one and six months, with total six-month healthcare costs of USD 246,717. By end of follow-up, 35.3% of SOT recipients died with a median time to death of 31 days.

## 4. Discussion

In this study of high-risk patients hospitalized for IA, including those with hematologic and non-hematologic malignancies, BMT/HCT and SOT, each cohort was found to have protracted total length-of-stay (12–18 days) and ICU stay (10–13 days) and costly (USD 79,058–USD 172,342) index IA hospital stays that resulted in death or need for hospice in 28.1–36.1% of patients. Among those surviving to discharge, more than 50% and up to two-thirds were re-admitted for any cause by six months, with six-month all-cause total treatment costs ranging from USD 213,378 to USD 397,857. At the end of follow-up, all-cause mortality in these at-risk populations was 33.6–40.6%. Patients with hematologic malignancies and BMT/HCT recipients had the longest LOSs and highest total index hospital and 6-month follow-up treatment costs, as well as the highest all-cause mortality rates at the end of follow-up.

While studies reporting on in-hospital outcomes and short-term re-admission rates associated with IA in high-risk subgroups have been published [4,5,6,7,9], there is a relative paucity of studies evaluating post-discharge resource use and costs in the literature. By comparison, our study provides important, contemporary data on both index IA hospitalization costs and total treatment costs and resource use beyond the index hospitalization.

Our study also provides the first comparative HCRU analysis of the four major cohorts at-risk for development of IA. The detailed comorbidities provided in Figure 1 illustrate the potential risks for IA, as well as the additional conditions that may incur HCRU. More than one-half of patients admitted for IA with hematological malignancies and BMT/HCT were neutropenic, which would incur costs of recombinant cytokines, such as rhu-G-CSF. Patients with SOT typically had approximately twice the frequency of diabetes mellitus, which would also add to HCRU. Patients with hematological malignancies, non-hematological malignancies, and HCT were also receiving antineoplastic therapy during their IA admissions, which would further add to HCRU, while patients with BMT/HCT and SOT recipients were receiving immunosuppressive agents for prevention or treatment of GVHD and allograft rejection, respectively. These costs are added to those of mold active triazoles for treatment of IA.

While patients with hematological malignancies, BMT/HCT, and SOT are well recognized as having risk factors for development of IA [10,11,12,13], our study also identified patients with non-hematological malignancies without transplantation. The potential risk factors in this patient population for development of IA included chemotherapy, neutropenia, and corticosteroids. This population also had a median of 10 days of ICU admission, further signifying serious illness among these patients, which has been associated with development of IA. The relatively high frequency of COPD in these patients may also have contributed to development of invasive pulmonary aspergillosis, particularly chronic pulmonary aspergillosis [14]. These risk factors for development of IA that were observed in our cohort of patients with non-hematological malignancies, were also reported by other investigators in similar populations [15,16,17].

The long and costly IA hospitalizations, and high inpatient mortality observed in IA patients with malignancy or transplantation in our study are generally consistent with findings from a retrospective study of Healthcare Cost and Utilization Project (HCUP), National Inpatient Sample (NIS) 2018 data by Rayens and colleagues [5]. The investigators found that in patients hospitalized with aspergillosis who had any type of malignancy, mean LOS was 18.0 days, hospital charges were USD 307,230, and inpatient mortality was 22.4%. Similarly, in those with a history of transplant, mean LOS was 14.6 days, hospital charges were USD 113,040 and inpatient mortality rate was 11.5%. While IA hospitalizations appeared more expensive and the mortality rate somewhat lower in Rayens et al. [5] versus our study, this may be explained by their reporting of charges (versus costs) and our study’s definition of mortality including the need for hospice.

Our study also found the IA patient cohorts with a history of BMT/HCT and hematologic malignancy were the most likely of the four cohorts to experience hospital re-admission at six months. In a retrospective study using US National Readmission Database (NRD) data (2013–2019) which reported all-cause re-admission rates in HCT patients with invasive pulmonary aspergillosis (IPA) (a group often comprised substantially of patients with hematologic malignancies), Khalil and colleagues found one-month re-admission rates of 36.2% [9]. While this re-admission estimate is higher than in our own BMT/HSCT or hematologic malignancy cohorts (up to 19.7%), it reinforces the high burden of illness associated with IA infections in such patients.

This study has several limitations. First, as with all claims database studies, misclassification bias is a concern [18,19]. IA admissions were assigned based on the presence of ICD-10 diagnosis codes. Clinical and/or laboratory data needed to support a more formal diagnosis or describe the severity of IA were not available in our data set to fulfill standard definitions of IA [20,21]. Second, while our study was modestly sized, it was not sufficiently large to run analyses of IA-specific HCRU or costs. Next, HCRU could have occurred outside of IQVIA’s data partners and therefore may not have been captured in our analysis [8]. Such missed data could have resulted in an underestimation of HCRU and costs. Lastly, because our study spanned a substantial time window, changes in the practice for managing IA, such as approval of new antifungal agents, improved diagnostics, and updated treatment guidelines, may have influenced our results.

In conclusion, hospitalizations for IA in patients with malignancies or transplantation are long, costly, and end with readmission, hospice, or death in more than one-third of patients. Novel antifungal agents with improved effectiveness against IA are needed to reduce time in hospital, complications, need for re-admission, and overall cost of care.

## Figures and Tables

**Table 1:** Clinical characteristics of patients with malignancies or transplantation that developed invasive aspergillosis *.

Clinical Characteristics	Hematologic Malignancy N = 317	Non-Hematologic Malignancy N = 155	BMT/HCT N = 133	SOT N = 173
	n (%)	n (%)	n (%)	n (%)
Hematologic malignancy	317 (100.0)	25 (16.1)	115 (86.5)	9 (5.2)
Leukemia	260 (82.0)	21 (13.5)	96 (72.2)	6 (3.5)
Non-Hodgkin lymphoma	71 (22.4)	5 (3.2)	21 (15.8)	2 (1.2)
Non-hematologic malignancy	25 (7.9)	155 (100.0)	6 (4.5)	10 (5.8)
Breast	9 (2.8)	25 (16.1)	3 (2.3)	1 (0.6)
Colon, rectum, anus	4 (1.3)	17 (11.0)	1 (0.8)	2 (1.2)
Lung, trachea, bronchus	8 (2.5)	84 (54.2)	0 (0.0)	2 (1.2)
Ovary, uterus, cervix	1 (0.3)	6 (3.9)	0 (0.0)	0 (0.0)
Pancreas	0 (0.0)	2 (1.3)	0 (0.0)	0 (0.0)
Prostate	2 (0.6)	11 (7.1)	1 (0.8)	3 (1.7)
Urinary	2 (0.6)	22 (14.2)	1 (0.8)	2 (1.2)
BMT/HCT history ^†^	110 (34.7)	6 (3.9)	125 (94.0)	6 (3.5)
BMT	67 (21.1)	4 (2.6)	77 (57.9)	4 (2.3)
HCT	100 (31.5)	6 (3.9)	111 (83.5)	4 (2.3)
SOT history ^†^	9 (2.8)	9 (5.8)	7 (5.3)	170 (98.3)
Heart	0 (0.0)	2 (1.3)	0 (0.0)	16 (9.3)
Heart and lung	0 (0.0)	0 (0.0)	0 (0.0)	3 (1.7)
Kidney	2 (0.6)	3 (1.9)	1 (0.8)	74 (42.8)
Liver	0 (0.0)	0 (0.0)	0 (0.0)	21 (12.1)
Lung	1 (0.3)	4 (2.6)	0 (0.0)	74 (42.8)
Other	6 (1.9)	1 (0.6)	6 (4.5)	17 (9.8)

BMT = bone marrow transplant; HCT = hematopoietic cell transplant; SOT = solid organ transplant. * The hematologic malignancy, non-hematologic malignancy, BMT/HCT, and SOT cohorts are not mutually exclusive. ^†^ Patients who did not have a transplantation history diagnosis code qualified for the analysis by having a transplantation complication code.

**Table 2:** Demographics of patients with malignancies or transplantation that developed invasive aspergillosis *.

Characteristics	Hematologic Malignancy N = 317	Non-Hematologic Malignancy N = 155	BMT/HCT N = 133	SOT N = 173
	n (%)	n (%)	n (%)	n (%)
Age group				
18–44 years	44 (13.9)	6 (3.9)	26 (19.6)	18 (10.4)
45–64 years	138 (43.5)	44 (28.4)	57 (42.9)	77 (44.5)
65+ years	135 (42.6)	105 (67.7)	50 (37.6)	78 (45.1)
Gender				
Female	116 (36.6)	73 (47.1)	52 (39.1)	62 (35.8)
Male	201 (63.4)	82 (52.9)	81 (60.9)	111 (64.2)
Geographic region ^†^				
Northeast	23 (7.3)	20 (12.9)	9 (6.8)	12 (6.9)
Midwest	31 (9.8)	13 (8.4)	11 (8.3)	4 (2.3)
South	36 (11.4)	30 (19.4)	5 (3.8)	64 (37.0)
West	227 (71.6)	92 (59.4)	108 (81.2)	93 (53.8)
Payer type				
Commercial	122 (38.5)	49 (31.6)	52 (39.1)	53 (30.6)
Medicaid	26 (8.2)	4 (2.6)	14 (10.5)	5 (2.9)
Medicare	111 (35.0)	87 (56.1)	43 (32.3)	96 (55.5)
Unknown	58 (18.3)	15 (9.7)	24 (18.1)	19 (11.0)

BMT = bone marrow transplant; HCT = hematopoietic cell transplant; SOT = solid organ transplant. * The hematologic malignancy, non-hematologic malignancy, BMT/HCT, and SOT cohorts are not mutually exclusive. ^†^ US Census regions.

**Table 3:** Index invasive aspergillosis admission length-of-stay and treatment costs and 1- and 6- month post-hospitalization follow-up treatment costs ^†^ in patients with history of malignancies and transplantation *.

HCRU/Costs (USD)	Hematologic Malignancy N = 317	Non-Hematologic Malignancy N = 155	BMT/HCT N = 133	SOT N = 173
Index IA Hospitalization				
LOS, days, median (Q1, Q3)	18 (9, 33)	12 (7, 20)	18 (8, 33)	15 (8, 28)
ICU LOS, days, median (Q1, Q3)	12 (5, 21)	10 (6, 16)	12 (5, 22)	13 (6, 23)
Total costs, 2023 USD, median (Q1, Q3)	USD 151,489 (USD 72,916, USD 372,649)	USD 79,058 (USD 43,016, USD 186,103)	USD 172,342 (USD 80,686, USD 507,860)	USD 124,753 (USD 54,999, USD 339,078)
All-Cause Total Treatment Costs During Follow-Up				
1 Month, 2023 USD, median (Q1, Q3)	USD 236,325 (USD 126,282, USD 520,513)	USD 117,438 (USD 62,439, USD 249,349)	USD 226,676 (USD 115,488, USD 599,601)	USD 132,624 (USD 70,155, USD 443,311)
6 Months, 2023 USD, median (Q1, Q3)	USD 397,857 (USD 237,651, USD 697,059)	USD 213,378 (USD 108,048, USD 420,493)	USD 389,296 (USD 172,248, USD 781,760	USD 246,717 (USD 130,457, USD 444,578)

BMT = bone marrow transplant; HCRU = healthcare resource utilization; HCT = hematopoietic cell transplantation; IA = invasive aspergillosis; ICU = intensive care unit; LOS = length-of-stay; Q = quartile; SOT = solid organ transplantation; USD = United States dollar. * The hematologic malignancy, non-hematologic malignancy, BMT/HCT, and SOT cohorts are not mutually exclusive. ^†^ Among patients with at least that duration of follow-up.

## Data Availability

Data for this study was obtained by license through IQVIA.
